# Efficacy and Safety of Salvage-line Nivolumab Monotherapy for Advanced Esophageal Squamous Cell Carcinoma: Comparison of 240 mg Versus 480 mg Doses

**DOI:** 10.1007/s12029-024-01092-w

**Published:** 2024-07-15

**Authors:** Yuko Murashima, Shun Yamamoto, Toshiharu Hirose, Toru Kadono, Go Ikeda, Akihiro Ohara, Mai Itoyama, Kazuki Yokoyama, Yoshitaka Honma, Koshiro Ishiyama, Jyunya Oguma, Hiroyuki Daiko, Ken Kato

**Affiliations:** 1 Department of Head and Neck, Esophageal Medical Oncology, National Cancer Center Hospital, 5-1-1 Tsukiji, Chuo-ku, Tokyo, 104-0045 Japan; 2 Department of Gastroenterology, St. Luke’s International Hospital, Tokyo, Japan; 3 Department of Gastrointestinal Medical Oncology, National Cancer Center Hospital, Tokyo, Japan; 4 Department of Esophageal Surgery, National Cancer Center Hospital, Tokyo, Japan; 5 Cancer Chemotherapy Center, Osaka Medical and Pharmaceutical University, Takatsuki, Osaka Japan; 6 Department of Gastroenterology, Nippon Medical School Graduate School of Medicine, Tokyo, Japan

**Keywords:** Esophageal cancer, Esophageal squamous cell carcinoma, Nivolumab, Chemotherapy

## Abstract

**Background:**

Nivolumab monotherapy is the standard second-line treatment for advanced esophageal squamous cell carcinoma (ESCC) after failure of platinum-based chemotherapy without anti-PD-1 antibody. Fixed dosing with 240 mg every 2 weeks was approved initially, followed by fixed dosing with 480 mg every 4 weeks based on pharmacokinetics data. However, information on the comparative efficacy and safety of the two doses remains limited.

**Methods:**

We compared progression-free survival (PFS), overall survival (OS), objective response rate (ORR), and the incidence of adverse events (AEs) between the two doses in 117 patients who received second-line (*n* = 85) or later-line (*n* = 32) nivolumab monotherapy at our institution between January 2016 and December 2021.

**Results:**

In the second-line group, patient characteristics for the 240 mg and 480 mg groups were as follows (240 mg vs. 480 mg): performance status (PS) 0/1/2 was 34/61/5% vs. 54/42/4%, and prior fluoropyrimidine plus platinum therapy (FP) was 81.3% vs. 42.3%. In the later-line group, the characteristics were: PS 0/1/2 was 28/60/12% vs. 14/86/0%, and prior FP was 60.0% vs. 42.8%. ORR was 11.9 vs. 24.0% in the second-line group (*p* = 0.19) and 0 vs. 14.3% in the later-line group (*p* = 0.22). Median PFS was 1.7 vs. 4.1 months on second-line (hazard ratio [HR] 0.60, 95% confidence interval [CI] 0.35–1.01, *p* = 0.056) and 1.4 vs. 1.8 months on later-line (HR 0.58, 95% CI 0.23–1.46, *p* = 0.25); AEs of any grade were observed in 58.3 vs. 69.7%, respectively.

**Conclusions:**

The efficacy and safety of the two doses of nivolumab monotherapy were comparable in patients with advanced ESCC.

## Introduction

The annual incidence of newly diagnosed esophageal cancer (EC) was estimated to be 26,382 in Japan in 2019, ranking 11th among all cancers [1], and to be 20,640 in the US in 2022, accounting for 1.1% of all cancers [2]. EC is classified histologically into squamous cell carcinoma (ESCC) and adenocarcinoma. ESCC has been reported to account for 87.8% of all EC and is more common in Japan than in Western countries [3]. Smoking and alcohol consumption are known to be major risk factors for ESCC, while Barrett's epithelium, reflux esophagitis, and high body mass index are known to be major risk factors for adenocarcinoma [4–7]. Fluoropyrimidine plus platinum combination therapy was traditionally considered to be first-line therapy for patients with advanced ESCC [8, 9]. However, more recently, fluoropyrimidine plus platinum, nivolumab, nivolumab plus ipilimumab, fluoropyrimidine plus platinum, and pembrolizumab have become established as first-line treatments based on the results of the CheckMate 648 and KEYNOTE-590 trials [10, 11]. Taxanes, nivolumab monotherapy, and pembrolizumab monotherapy can also be used as second-line therapy [12–14]. In the 2022 Japanese guidelines for EC, nivolumab monotherapy is strongly recommended for ESCC with no prior anti-PD-L1 therapy and pembrolizumab monotherapy is weakly recommended for ESCC with a combined positive score of ≥ 10 and no prior anti-PD-L1 therapy or microsatellite instability-high or tumor mutational burden-high with no prior anti-PD-L1 antibody. Paclitaxel is weakly recommended for patients with no history of taxane use with or without prior anti-PD-L1 antibody therapy.

Nivolumab was initially approved at a fixed dosage of 240 mg every 2 weeks (Q2W) in February 2020 based on the results of the ATTRACTION-3 trial [12]. A fixed dosage of 480 mg every 4 weeks (Q4W) became available in September 2020 based on pharmacokinetics data [15]. However, there is little clinical information on the difference of efficacy and safety between two dosages in patients with advanced ESCC.

## Methods

### Design and Patients

This study had a single-center, retrospective design and analyzed data from patients with advanced ESCC treated with second-line or later-line nivolumab monotherapy at the National Cancer Center Hospital in Japan between January 2016 and December 2021. Performance status (PS) was assessed according to the Eastern Cooperative Oncology Group criteria, and the eighth edition of the Union for International Cancer Control TNM classification was used for cancer staging. This study was approved by the National Cancer Center Hospital’s institutional review board (approval number 2020–287) and conducted in accordance with the ethical principles outlined in the Declaration of Helsinki. Although informed consent was not obtained, patients were provided with the opportunity to opt out.

### Assessments

We compared the objective response rate (ORR), progression-free survival (PFS), and adverse event (AE) rate between the fixed dosage of 240 mg Q2W and the fixed dosage of 480 mg Q4W in the second-line or later-line setting. The ORR was defined as the proportion of patients with a complete or partial response to treatment according to RECIST (Response Evaluation Criteria in Solid Tumors) version 1.1. The response was evaluated on computed tomography scans obtained every 2–3 months. Overall survival was defined as the interval between the date of initiation of treatment and death (from any cause) or censored at the last date of confirmed survival. PFS was defined as the interval between the start of treatment and the date of the first documentation of disease progression or death (from any cause), whichever occurred first, or was censored at the last date of confirmed survival without disease progression. AEs were graded according to the CTCAE (Common Terminology Criteria of Adverse Events) version 5.0.

### Statistical Analysis

The median follow-up duration was estimated using the reversed Kaplan-Meier method. Survival curves were drawn using Kaplan-Meier methods, and these difference between groups was evaluated using the log-rank test. Differences in the distribution of ordinal variables were analyzed using the Chi-squared test or Fisher's exact test. All statistical analyses were performed using EZR (Saitama Medical Center, Jichi Medical University, Saitama, Japan) [16]. A *p*-value of < 0.05 was considered statistically significant.

## Results

### Patient Characteristics

**Fig. 1:**
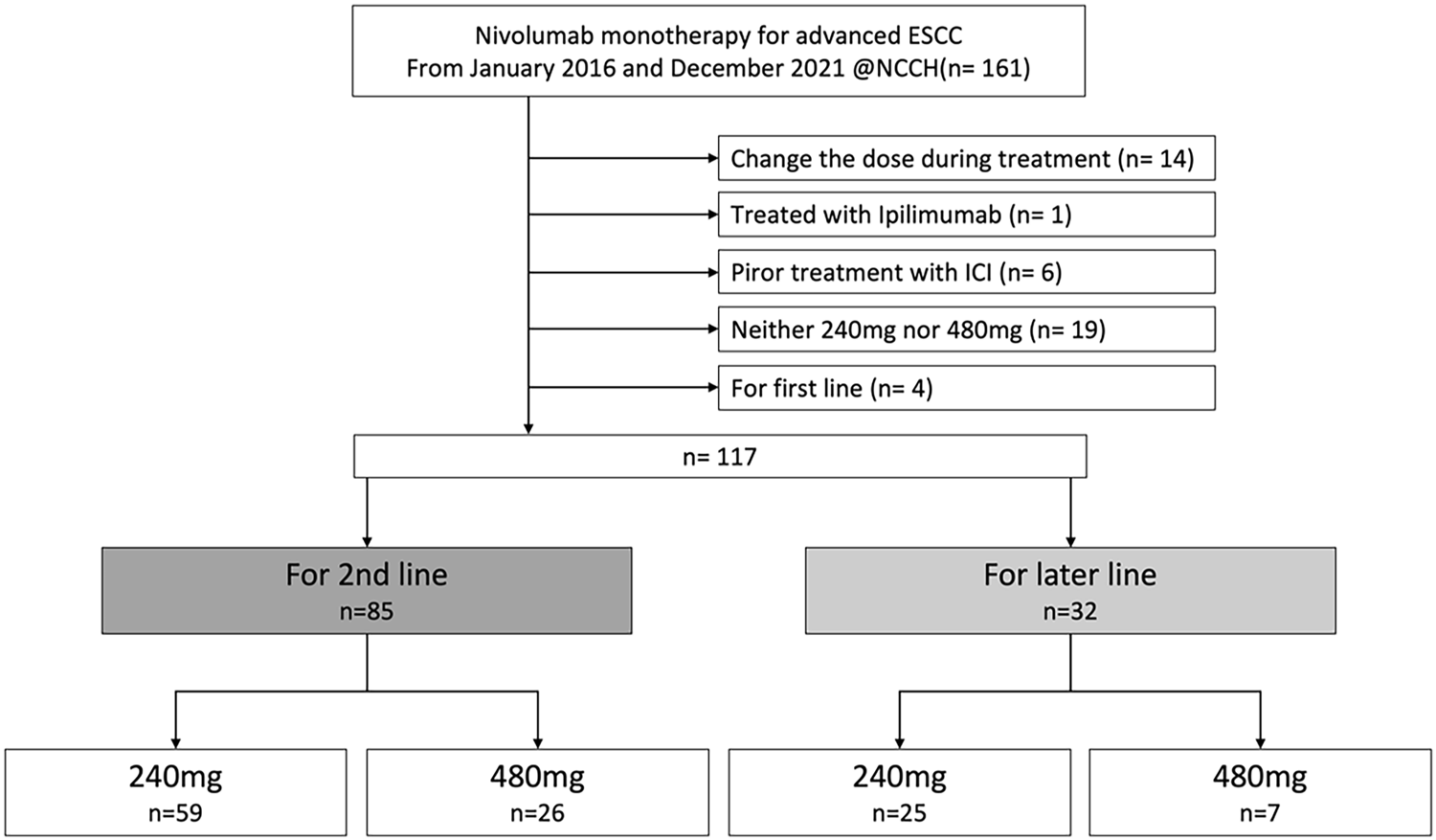
CONSORT diagram showing the patient selection process. ESCC, esophageal squamous cell carcinoma

In total, 117 patients with ESCC received nivolumab monotherapy between January 2016 and December 2021. Of the 85 patients who received nivolumab monotherapy as a second-line treatment, 59 received 240 mg Q2W and 26 received 480 mg Q4W. Of the remaining 32 patients who received nivolumab monotherapy as a later-line treatment, 25 received 240 mg Q2W and 7 received 480 mg Q4W (Fig. 1). Patients who switched from 240 mg Q2W to 480 mg Q4W during treatment were excluded. The patient characteristics are shown in Tables 1 and 2. In the second-line group, the median age was 68 (range, 46–85) years in the patients who received 240 mg Q2W and 68.5 (range, 51–84) years in those who received 480 mg Q4W. PS was 0, 1 and 2, respectively, in 34%, 61%, and 5% of patients who received 240 mg Q2W and in 54%, 42%, and 4% of those who received 480 mg Q4W. There was a history of FP therapy in 81.3% of those who received 240 mg Q2W and in 42.3% of those who received 480 mg Q4W. In the later-line group, the median age was 63.5 (range, 47–85) years in the patients who received 240 mg Q2W and 58 (range, 46–80) years in those who received 480 mg Q4W. PS was 0, 1 and 2, respectively, in 28%, 60%, and 12% of patients who received 240 mg Q2W and 14%, 86%, and 0% in those who received 480 mg Q4W. There was a history of FP therapy in 60.0% of patients who received 240 mg Q2W and in 42.8% of those who received 480 mg Q4W. The backgrounds between the 240 mg Q2W and 480 mg Q4W fixed dosages of nivolumab were found to have no significant differences.

**Table 1:** Background characteristics in patients receiving nivolumab as second-line monotherapy for advanced esophageal squamous cell carcinoma

**Variable**	**240 mg Q2W (*n*** **= 59)**	**480 mg Q4W (*n*** **= 26)**	***p*-value**
Age, years, median (range)	68 (46–85)	68.5 (51–84)	0.712
Male sex, number_(percentage)	52 (88.1)	22 (84.6)	0.730
ECOG PS 0, number_(percentage)	20 (33.9)	14 (53.8)	0.234
ECOG PS 1, number_(percentage)	36 (61.0)	11 (42.3)	
ECOG PS 2, number_(percentage)	3 (5.1)	1 (3.8)	
Recurrence, number_(percentage)	33 (55.9)	16 (61.5)	0.812
Organs with metastases, median (range)	2 (1–6)	2 (0–5)	0.769
Prior chemotherapy; FP, number_(percentage)	48 (81.3)	11 (42.3)	**< 0.05**
Prior chemotherapy; DCF, number_(percentage)	11 (18.6)	15 (57.7)	
Prior radiotherapy, number_(percentage)	45 (76.3)	15 (57.7)	0.120
Prior surgery, number_(percentage)	28 (47.5)	18 (69.2)	0.098

*ECOG* Eastern Cooperative Oncology Group, *PS* performance status

**Table 2:** Background characteristics in patients receiving nivolumab as later-line monotherapy for advanced esophageal squamous cell carcinoma

**Variable**	**240 mg Q2W (*n*** **= 25)**	**480 mg Q4W (*n*** **= 7)**	***p*-value**
Age, years, median (range)	63.5 (47–85)	58 (46–80)	0.661
Male sex, number_(percentage)	21 (84)	6 (85.7)	1
ECOG PS 0, number_(percentage)	7 (28)	1 (14.3)	0.536
ECOG PS 1, number_(percentage)	15 (60)	6 (85.7)	
ECOG PS 2, number_(percentage)	3 (5.1)	1 (3.8)	
Recurrence, number_(percentage)	9 (36)	1 (14.3)	0.387
Organs with metastases, median (range)	2 (1–6)	2 (0–5)	0.769
Prior chemotherapy; FP, number_(percentage)	15 (60)	3 (42.8)	0.669
Prior chemotherapy; DCF, number_(percentage)	10 (40)	4 (57.1)	
Prior radiotherapy, number_(percentage)	24 (96)	5 (71.4)	0.113
Prior surgery, number_(percentage)	9 (36)	1 (14.3)	0.387

*ECOG* Eastern Cooperative Oncology Group, *Q2W* fixed dosage every two weeks, *Q4W* fixed dosage every four weeks, *PS* performance status

### Efficacy

**Fig. 2:**
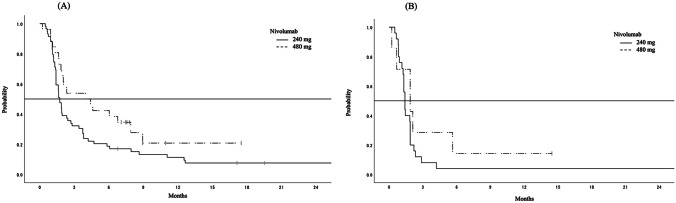
**A** Progression-free survival in patients receiving nivolumab as second-line monotherapy for advanced esophageal squamous cell carcinoma. The median follow-up duration was 10.3 months in the 240 mg Q2W group and 8.1 months in the 480 mg Q4W group; median progression-free survival was 1.7 months (95% CI 1.38–2.30) and 4.1 months (95% CI 1.81–7.79), respectively (hazard ratio 0.60, 95% CI 0.35–1.01, *p* = 0.056). CI, confidence interval; Q2W, fixed dosage every two weeks; Q4W, fixed dosage every four weeks. **B** Progression-free survival in patients receiving nivolumab as later-line monotherapy for advanced esophageal squamous cell carcinoma. The median follow-up duration was 4.0 months in the 240 mg Q2W group and 5.8 months in the 480 mg Q4W group; median progression-free-survival was 1.4 months (95% CI 1.22–1.84) and 1.8 months (95% CI 0.23–5.55), respectively (hazard ratio 0.58, 95% CI 0.23–1.46, *p* = 0.251). CI, confidence interval; Q2W, fixed dosage every two weeks; Q4W, fixed dosage every four weeks

**Fig. 3:**
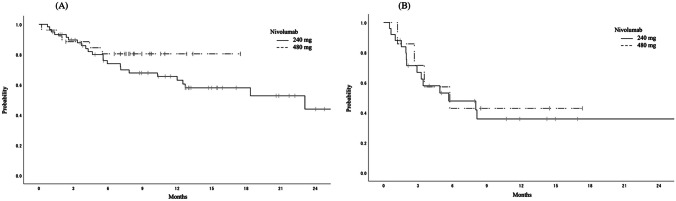
**A** Overall survival in patients receiving nivolumab as second-line monotherapy for advanced esophageal squamous cell carcinoma. The median overall survival was 22.8 months (95% CI 11.8-NA) and NA, respectively (hazard ratio 0.99, 95% CI 0.99–1.00, *p* = 0.26). NA, not available; CI, confidence interval; Q2W, fixed dosage every two weeks; Q4W, fixed dosage every four weeks. **B** Overall survival in patients receiving nivolumab as later-line monotherapy for advanced esophageal squamous cell carcinoma. The median overall survival was 5.6 months (95% CI 1.98-NA) and 5.7 months (95% CI 1.18-NA), respectively (hazard ratio 0.99, 95% CI 0.99–1.00, *p* = 0.75). NA, not available; CI, confidence interval; Q2W, fixed dosage every two weeks; Q4W, fixed dosage every four weeks

In the second-line group, 62 patients had measurable lesions according to RECIST version 1.1 at the start of treatment. The ORR was 11.9% in patients who received 240 mg Q2W and 24.0% in those who received 480 mg Q4W (*p* = 0.193). In the later-line group, there were 21 patients. The ORR was 0% in patients who received 240 mg Q2W and 14.3% in those who received 480 mg Q4W (*p* = 0.219). The median follow-up duration was 10.3 months in patients who received 240 mg Q2W and 8.1 months in those who received 480 mg Q4W; median PFS was 1.7 months and 4.1 months, respectively (hazard ratio [HR] 0.60, 95% confidence interval [CI] 0.35–1.01; *p* = 0.056) (Fig. 2A). In the later-line group, the median follow-up duration was 4.0 months in patients who received 240 mg Q2W and 5.8 months in those who received 480 mg Q4W; median PFS was 1.4 months and 1.8 months, respectively (HR 0.58, 95% CI 0.23–1.46; *p* = 0.25) (Fig. 2B). In the second-line group, the median OS was 22.7 months in patients who received 240 mg Q2W as compared with Not Available (NA) in those who received 480 mg Q4W (HR 1.00, 95% CI 11.8-NA; *P* = 0.25) (Fig. 3A). In the later-line group, the median OS was 5.6 months in patients who received 240 mg Q2W as compared with 5.7 months in those who received 480 mg Q4W (HR 1.00, 95% CI 1.97-NA; *P* = 0.753) (Fig. 3B). There was no significant difference in ORR, PFS, or OS between the fixed dosages of 240 mg Q2W and 480 mg Q4W in either treatment line.

### Safety

Safety was compared between the dosage regimens independent of treatment line. Treatment-related AEs of any grade were observed in 58.3% of patients in the 240 mg Q2W group and in 69.7% of those in the 480 mg Q4W group (Table 3). The frequencies of grade 1, 2, 3, and 4 AEs were as follows: rash, 11.9%, 4.8%, 0%, and 0%, respectively, in the 240 mg Q2W group and 3.0%, 3.0%, 0%, and 0% in the 480 mg Q4W group; pruritus, 14.3%, 6.0%, 0%, and 0% in the 240 mg Q2W group and 12.1%, 0%, 0%, and 0% in the 480 mg Q4W group; diarrhea, 3.6%, 4.8%, 1.2%, and 0% in the 240 mg Q2W group and 3.0%, 3.0%, 0%, and 0% in the 480 mg Q4W group; and interstitial pneumonia, 0%, 0%, 3.6%, and 0% in the 240 mg Q2W group and 0%, 9.1%, 6.1%, and 0% in the 480 mg Q4W group. There were no treatment-related deaths in either dosage group.

**Table 3:** Adverse events of any grade according to dose strength

**Adverse event**	**240 mg Q2W (*n* = 84)**				**480 mg Q4W (*n* = 33)**			
	Grade 1	Grade 2	Grade 3	Grade 4	Grade 1	Grade 2	Grade 3	Grade 4
Rash	10 (11.9)	4 (4.8)	0	0	1 (3.0)	1 (3.0)	0	0
Pruritus	12 (14.3)	5 (6.0)	0	0	4 (12.1)	0	0	0
Thyroid dysfunction	1 (1.2)	1 (1.2)	0	0	0	2 (6.1)	0	0
Hyponatremia	1 (1.2)	0	0	1 (1.2)	0	0	1 (3.0)	0
Diarrhea	3 (3.6)	4 (4.8)	1 (1.2)	0	1 (3.0)	1 (3.0)	0	0
Anemia	1 (1.2)	1 (1.2)	2 (2.4)	0	3 (9.1)	0	0	0
Elevated hepatic enzymes	0	1 (1.2)	1 (1.2)	0	0	0	1 (3.0)	0
Adrenal insufficiency	1 (1.2)	2 (2.4)	0	0	0	1 (3.0)	1 (3.0)	0
Interstitial pneumonia	0	0	3 (3.6)	0	0	3 (9.1)	2 (6.1)	0
Pneumonia	0	2 (2.4)	4 (4.8)	0	0	1 (3.0)	3 (9.1)	0

Data are presented as the number (%). There were no treatment-related deaths

*Q2W* fixed dosage every two weeks, *Q4W* fixed dosage every four weeks

## Discussion

This study found no statistically significant differences in efficacy and safety between the 240 mg Q2W and 480 mg Q4W fixed dosages of nivolumab when used as salvage-line monotherapy for advanced ESCC. Nivolumab monotherapy was first approved at a dosage of 2 mg/kg every 3 weeks for melanoma based on the results of a Phase II trial [17]. Next, a dosage of 3 mg/kg every 2 weeks was approved for lung cancer based on the results of a Phase III trial [18]. Subsequently, a fixed dosage of 240 mg Q2W became available based on population pharmacokinetics and exposure-response analyses showing comparability of exposure, safety, and efficacy between dosages of 3 mg/kg every 2 weeks and a fixed dosage of 240 mg Q2W [19]. Finally, a fixed dosage of 480 mg Q4W was approved based on modeling and simulation showing that the benefit-risk of a fixed dosage of 480 mg Q4W was a similar to that of 3 mg/kg Q2W [2]. In this study, since the approval of 480 mg Q4W regimen, 480 mg Q4W regimen has been predominantly chosen.

A retrospective cohort study of nivolumab as an adjuvant treatment for melanoma compared the duration of therapy and safety of four different dosage (de novo nivolumab 480 mg Q4W, switched to nivolumab 480 mg Q4W after nivolumab 240 mg or 3 mg/kg Q2W, de novo nivolumab 3 mg/kg Q2W, and de novo nivolumab 240 mg Q2W) [20]. The safety profiles of nivolumab 240 mg Q2W and 480 mg Q4W were similar and comparable with the safety profile of nivolumab 3 mg/kg Q2W [20]. In the present study, ORR was 11.9% in patients who received 240 mg Q2W and 24.0% in those who received 480 mg Q4W (*p* = 0.193) in the second-line setting and 0% and 14.3%, respectively (*p* = 0.219) in the later-line setting; the difference according to treatment line was not statistically significant. Median PFS was 1.7 months in patients who received 240 mg Q2W and 4.1 months in those who received 480 mg Q4W as a second-line treatment (HR 0.60, 95% CI 0.35–1.01; *p* = 0.056) and 1.4 months and 1.8 months, respectively, in those who received nivolumab as a later-line treatment (HR 0.58, 95% CI 0.23–1.46; *p* = 0.25). Although there was no statistically significant difference between the two fixed dosages, 480 mg Q4W showed a trend of slightly better efficacy. This finding may reflect differences in patient background characteristics or differences in the timing of standard treatment. This is the first study to demonstrate the efficacy and safety of nivolumab at these dosages for advanced ESCC, and its findings are similar to those in melanoma and other solid and hematological tumors [20, 21].

Our finding of no significant difference in AEs between the two dosages of nivolumab is consistent with a previous report [22]. Interstitial pneumonia cases tended to be more frequent in patients who received 480 mg Q4W. However, chart review revealed that patients who developed interstitial pneumonia had pre-existing poor lung function because of underlying collagen disease or chronic obstructive pulmonary disease. When there was evidence of pneumonia and an increased oxygen demand, there was a tendency to administer steroids early before the pneumonia became severe. This could reflect implementation of methods to manage immune-related AEs potentially caused by nivolumab.

This study has some limitations. First, it had a single-center retrospective cohort design and a small number of patients. In the second-line cohort of the 480 mg group, there was a trend towards improved PFS and OS; however, this did not reach statistical significance. The study may have been underpowered to detect small differences, potentially resulting in a Type II error. Second, it included a number of patients with poor PS who were started on nivolumab third-line or later-line treatment immediately after the drug received regulatory approval for use in this setting. In several studies of nivolumab in patients with various types of cancer, subgroup analyses in Japanese patients have identified more cases with good PS and an association between PS and overall survival [17, 18, 23]. Patients with better PS are more likely to receive further treatment. Our present findings indicate that the efficacy and safety of the two currently approved fixed dosages of nivolumab were comparable when used as monotherapy in patients with advanced ESCC. Moreover, they are based on real-world data and confirm that the dosages of nivolumab that have been used up to now are safe and effective.

In conclusion, the two fixed dosages of nivolumab monotherapy currently approved for patients with advanced ESCC were comparable in terms of efficacy and safety.

## Data Availability

Individual participant data underpinning the results reported in this article will be shared, after de-identification, with investigators whose proposed data usage has been approved. Proposals for data access should be directed to kenkato@ncc.go.jp.
